# Anti-Inflammatory Protein Isolated from Tamarind Promotes Better Histological Aspects in the Intestine Regardless of the Improvement of Intestinal Permeability in a Preclinical Study of Diet-Induced Obesity

**DOI:** 10.3390/nu14214669

**Published:** 2022-11-04

**Authors:** Mayara S. R. Lima, Catarina Gonçalves, Mafalda D. Neto, Maria Helena Macedo, Jaluza L. C. de Queiroz, Valéria C. da Silva, Izael de S. Costa, Christina da S. Camillo, Pedro Paulo de A. Santos, Aldo A. M. Lima, Lorenzo Pastrana, Bruna L. L. Maciel, Ana Heloneida A. Morais

**Affiliations:** 1 Postgraduate Program in Biochemistry and Molecular Biology, Biosciences Center, Federal University of Rio Grande do Norte, Natal 59075-000, RN, Brazil; 2 International Iberian Nanotechnology Laboratory, 4715-330 Braga, Portugal; 3 Postgraduate Program in Development and Technological Innovation in Medicines, Health Sciences Center, Federal University of Rio Grande do Norte, Natal 59075-000, RN, Brazil; 4 Nutrition Course, Potiguar University, Natal 59056-000, RN, Brazil; 5 Department of Morphology, Federal University of Rio Grande do Norte, Natal 59075-000, RN, Brazil; 6 Department of Physiology and Pharmacology, Federal University of Ceará, Fortaleza 60430-275, CE, Brazil; 7 Postgraduate Program in Nutrition, Health Sciences Center, Federal University of Rio Grande do Norte, Natal 59075-000, RN, Brazil; 8 Department of Nutrition, Federal University of Rio Grande do Norte, Natal 59075-000, RN, Brazil

**Keywords:** inflammation, intestinal mucosa, human neutrophil elastase, obesity, tumor necrosis factor-alpha

## Abstract

Obesity is associated with metabolic and physiological effects in the gut. In this study, we evaluated the anti-inflammatory effect of trypsin inhibitor isolated from tamarind seeds (TTI) in vitro (interaction with lipopolysaccharide (LPS) and inhibitory activity against human neutrophil elastase (HNE)), and using intestinal co-cultures of Caco-2:HT29-MTX cell lines inflamed with TNF-α (50 ng/mL) and a Wistar rat model of diet-induced obesity (n = 15). TTI was administered to animals by gavage (10 days), and the treated group (25 mg/kg/day) was compared to animals without treatment or treated with a nutritionally adequate diet. In the in vitro study, it showed inhibitory activity against HNE (93%). In co-cultures, there was no protection or recovery of the integrity of inflamed cell monolayers treated with TTI (1.0 mg/mL). In animals, TTI led to lower plasma concentrations of TNF-α and IL-6, total leukocytes, fasting glucose, and LDL-c (*p* < 0.05). The intestines demonstrated a lower degree of chronic enteritis, greater preservation of the submucosa, and greater intestinal wall thickness than the other groups (*p* = 0.042). Therefore, the better appearance of the intestine not reflected in the intestinal permeability added to the in vitro activity against HNE point to possibilities for new studies and applications related to this activity.

## 1. Introduction

The worldwide prevalence of obesity has been increasing over the last few years at epidemic proportions. According to the World Obesity Federation [1], by 2025, more than 16% of the world’s adult population will be obese. Obesity is a preventable condition caused by an energy imbalance between consumed and expended calories, mainly due to physical inactivity and increased intake of energy-dense foods, high in fat and sugar [2,3]. These excess calories can cause chronic low-grade inflammation, as adipocyte hypertrophy in obesity results in increased infiltration of macrophages into adipose tissue, which secrete inflammatory cytokines, such as tumor necrosis factor-alpha (TNF-α) and interleukin 6 (IL-6) [4,5].

In this context, as part of the treatment of obesity, it is important to have a nutritionally adequate diet because, while meals with high levels of glucose and fat can induce postprandial inflammation and low-grade chronic inflammation, healthy eating patterns are associated with lower concentrations of inflammatory markers [6].

Among these inflammatory markers, TNF-α has a central role since, despite initiating signal transduction pathways that lead to various cellular responses, including cell survival, differentiation, and proliferation, inadequate or excessive activation of its signaling is associated with chronic inflammation and can eventually lead to the development of pathological complications [7,8]. When in excess, one of the harmful actions of this cytokine in obese individuals is the alteration of the tight junctions (TJ) of the intestinal epithelial barrier, formed by proteins, such as occludins, claudins, and zonula occludens (ZO), which are essential in the regulation of intestinal permeability. Thus, despite having a variety of beneficial functions in the intestine, in some conditions, TNF-α can increase epithelial permeability, causing dysfunction in the intestinal barrier [9,10,11,12].

Changes in the integrity of this barrier facilitate the translocation of harmful substances and pathogens into the bloodstream, which is associated with the pathophysiology of several disorders other than obesity, such as diabetes, inflammatory bowel diseases, irritable bowel syndrome, celiac disease, food allergies, depression, and schizophrenia [13].

Given the importance of the intestinal barrier and aspects related to the development of its dysfunction, it is necessary to study components that act to protect or restore the integrity and functionality of this barrier. When these studies aim to verify the effectiveness of new molecules with therapeutic potential for humans, their validation is expected in in vitro cultures of intestinal cells that mimic the intestinal epithelium, such as Caco-2 and HT29-MTX cells, and also in animal models in which inflammatory bowel disorders are induced [14].

Some treatments studied aim to promote the recovery of intestinal integrity using anti-TNF-α therapy in patients with inflammatory bowel diseases. This is explained by the anti-inflammatory nature of the drugs used, consisting of anti-TNF-α antibodies. However, these drugs can have serious side effects, such as infections and autoimmune complications, as well as long-term reduction of therapeutic effect (Refs [15,16], demanding research on new therapeutic options. Therefore, several proteins and peptides have been exploited as therapeutics for intestinal inflammation, proving to reduce TNF-α and alleviate the condition of the studied diseases, improving macroscopic, histological, and permeability aspects in the intestine of mice and rats [17].

These proteins and peptides reduce TNF-α by acting mainly on the TNF-TNFR2 and TLR4-MD2 complex signaling pathways and, consequently, on the NF-κB signaling pathway [17]. There is also a description of the action of peptides and proteins that reduce intestinal inflammation by inhibiting serine proteases, such as trypsin and human neutrophil elastase (HNE), involved in the infiltration of leukocytes in the intestine [18]. Some proteins reduce TNF-α by acting as antioxidant enzymes, such as superoxide dismutase 1 (SOD1), since reactive oxygen species (ROS) play an essential role in intestinal inflammation [19].

It is still important to highlight that there are reports of the action of peptides that have anti-inflammatory effects on the intestinal mucosa by improving the intestinal microbiota composition with antimicrobial and/or immunomodulatory activities [20,21,22,23], either by neutralization or by interaction/binding to surface lipopolysaccharide (LPS) of the bacterial membrane [24,25].

Bioactive proteins obtained from the tamarind (*Tamarindus indica* L.) seed have been studied in the context of intestinal inflammation. For example, a protein identified as a trypsin inhibitor isolated from tamarind seeds (TTI) showed anti-inflammatory properties, leading to lower plasma concentrations of TNF-α and leptin in an animal model of obesity [26,27,28], in addition to HNE inhibition [29]. PurifiedTTI (TTIp) also promoted benefits regarding the histopathological aspects of the intestinal villi of Wistar rats fed with a high glycemic index and high glycemic load diet [30]. Thus, the aim of this work was to evaluate the effect of TTI on hematological, biochemical, and inflammatory parameters and the integrity and functionality of the intestinal barrier in a culture of inflamed intestinal cells and in an experimental model of obesity.

## 2. Materials and Methods

### 2.1. Obtaining the Trypsin Inhibitor Isolated from Tamarind Seeds (TTI)

Tamarind (*Tamarindus indica* L.) was botanically identified by the Brazilian Institute for the Environment and Renewable Natural Resources (IBAMA, Natal, RN, Brazil) and registered in the National System for the Management of Genetic Heritage and Associated Traditional Knowledge (SisGen) under the number AF6CE9C.

The extraction procedures, protein fractionation, and isolation of the tamarind trypsin inhibitor were performed according to Carvalho et al. [27]. All reagents used were of analytical grade and were obtained from Sigma-Aldrich^®^ (St. Louis, MI, USA) and from Vetec Química Fina Ltd.a (Rio de Janeiro, Brazil). The fruit was peeled and the pulp was removed to obtain the seeds, which were also peeled using stainless steel pliers. The cotyledons were processed in a grinder refrigerated at 6 °C and the powder was sieved through a 40 mesh to obtain a fine-grained flour.

The antitryptic activity was evaluated using 1.25 mM N-benzoyl-DL-arginine-p-nitroanilide (BApNA) as substrate [31], in all isolation steps, using 100 µL of crude protein extract (extracted in TRIS-HCl 50 mM, pH 7.5), protein fractions (fractionated by precipitation in ammonium sulfate in three saturation ranges: 0–30%, 30–60%, and 60–90%), and proteins retained in Trypsin-Sepharose CNBr 4B affinity chromatography. Protein quantification was performed according to Bradford [32], using bovine serum albumin (BSA) as a standard. The results of the specific trypsin inhibition activity were calculated by comparing the residual activity of the enzyme with the hydrolysis promoted in the absence of the inhibitor (100% of enzymatic activity). The specific trypsin inhibition activity value was expressed in inhibition units per milligram (IU/mg) of soluble proteins, where 1 IU is equivalent to a reduction of 0.01 in absorbance at 410 nm in the activity assay.

To determine the degree of purity and estimate the molecular mass of the inhibitor, electrophoresis was performed in a 12.5% polyacrylamide gel (SDS-PAGE), according to the methodology of Laemmli [33], using a molecular mass marker (GERPN756E, Amersham™ ECL™ Rainbow™ Marker-Full range).

### 2.2. In Vitro Interaction of TTI with Bacterial LPS

The interaction of TTI with bacterial LPS was investigated by incubating a constant amount (12.5 μg) of LPS from *Escherichia coli* O55:B5 (Sigma-Aldrich^®^, St. Louis, MI, USA) with three amounts of TTI (5.0; 7.5 or 10.0 μg) at 37 °C for 15 min. Subsequently, to determine whether TTI interacted with LPS, a SDS-PAGE (12.5%) was performed, according to the methodology of Laemmli [33], in which the electrophoresis gel was subjected to a current of 26 mA. As controls for the electrophoretic profile, TTI (10 μg) and LPS (12.5 μg) were applied separately to the gel, after being incubated alone and also without being subjected to incubation. The gel was silver stained according to Oakley, Kirsh, and Morris [34].

### 2.3. Inhibitory Activity against Human Neutrophil Elastase (HNE)

For the HNE inhibition assay, methoxysuc-cinyl-Ala-Ala-Pro-Val-p-nitroaniline (MeOSucAAPVpNA) was used as substrate [35]. A measure of 10 µL of leukocyte elastase solution (0.4 µg/µL) in phosphate-buffered saline (PBS, 150 mM, pH 7.4) was pre-incubated with 50 µL of TTI (30 µg) and 680 µL of buffer (PBS, 150 mM, pH 7.4) for 15 min at 37 °C. Then, the reaction was started with the addition of 5 µL of MeOSucAAPVpNA substrate at 5.0 × 10^−3^ M. After 2 h, the reaction was stopped with the addition of 250 µL of 2% citric acid. The tubes were centrifuged for 10 min (10,000× *g*) at 25 °C and the absorbance was measured in a spectrophotometer at 405 nm. The results are expressed in percentage of inhibition, in relation to the control of the enzyme without inhibitor (100% of enzymatic activity).

### 2.4. In Vitro Cellular Assays

#### 2.4.1. Cell Cultures

Cells were maintained in culture medium supplemented with fetal bovine serum (FBS) (PAN-Biotech GmbH, DE, EU), 100 U/mL of penicillin, and 100 µg/mL of streptomycin (1% Pen/Strep) and incubated at 37 °C in a humidified atmosphere with 5% CO_2_. The culture medium was changed every 2–3 days and the cells were routinely sub-cultured, ensuring a maximum confluence of 70–80%. To detach adherent cells, cultures were incubated for 5–10 min with trypsin-EDTA (0.25% trypsin; 0.1% EDTA).

#### 2.4.2. Caco-2

Caco-2 cells (passages 25–40), clone HTB-37™, from human colon adenocarcinoma, were obtained from the American Type Culture Collection (ATCC^®^) and cultured in Minimal Essential Medium (MEM) (PAN-Biotech GmbH, DE, EU) supplemented with 20% fetal bovine serum (FBS).

#### 2.4.3. HT29-MTX

Mucus-secreting cells HT29-MTX-E12 (passages 60–65) were obtained from the European Collection of Authenticated Cell Cultures (ECACC) and cultured in Dulbecco’s Modified Eagle Medium (DMEM) (PAN-Biotech GmbH, DE, EU) supplemented with 10% FBS.

#### 2.4.4. Semi-Confluent Cultures of Caco-2 Cells

Caco-2 cells were seeded at a density of 1.1 × 10^4^ cells/cm^2^ in 96-well plates and incubated for 24 h. The culture medium was removed and replaced by the samples to be tested, diluted in MEM (10%, *v*/*v*), to carry out cell viability experiments.

#### 2.4.5. Differentiated Monolayers of Caco-2:HT29-MTX Cells

Caco-2:HT29-MTX co-cultures, at a ratio of 9:1, were seeded at a density of 1.0 × 10^5^ cells/cm^2^ on a semipermeable membrane of inserts in 12-well plates, and cultured for 21 days in MEM to form a differentiated monolayer. On the 21st day, the culture medium was removed and replaced by the samples to be tested, diluted in MEM (10%, *v*/*v*).

#### 2.4.6. Cell Metabolic Activity

Cell metabolic activity was determined by the resazurin (RZ) reduction assay. Semi-confluent Caco-2 cells grown in 96-well plates were used in a first screening assay to evaluate the effect of TTI samples at three concentrations, based on the study by Oliveira [36] (0.1, 0.3, and 1.0 mg/mL) on cell metabolic activity. Then, the same concentrations were tested on the differentiated monolayers of Caco-2:HT29-MTX in 96-well plates.

After incubation with samples (4 h for semi-confluent cultures and 24 h for differentiated co-cultures), the culture medium was removed and replaced by resazurin sodium salt solution diluted in complete MEM (10% *v*/*v*), to a final concentration of 10.0 µg/mL, and the cultures were incubated for 4 h at 37 °C. MEM was used as a positive control and DMSO (40% *v*/*v*) was used as a negative control. Sterile water diluted in MEM (10%, *v*/*v*) as a control of the treatments.

The cell metabolic activity was determined by measuring the fluorescence of the product of resazurin reduction, resofurin (λex = 560 nm; λem = 590 nm), using a BioTeK^®^ Synergy H1 microplate reader (Winnoski, VT, USA). Results were expressed as a percentage of cell metabolic activity in relation to the positive control (cells in MEM).

#### 2.4.7. Intracellular Production of Reactive Oxygen Species (ROS)

The intracellular production of reactive oxygen species (ROS) by the differentiated co-cultures of Caco-2:HT29-MTX was determined in 96-well plates by the 2’,7’-dichlorofluorescein diacetate (DCFH-DA) assay. On the 21st day of differentiation, the culture medium was removed and replaced by 10 µM of DCFH-DA (Sigma-Aldrich^®^, St. Louis, MI, USA) in HBSS (Sigma-Aldrich^®^, St. Louis, MI, USA). After 1 h, the DCFH-DA solution was removed and the samples were added, diluted in HBSS (10% *v*/*v*), for final TTI concentrations of 0.1, 0.3, and 1.0 mg/mL. After 4 h of incubation at 37 °C, oxidative activity was determined by measuring dichlorofluorescein (DCF) fluorescence (λex = 495 nm; λem = 525 nm), using a BioTeK^®^ Synergy H1 microplate reader (Winnoski, VT, USA). 6-Hydroxy-2,5,7,8-tetramethylchromane-2-carboxylic acid (Trolox, 50 µg/mL) (Sigma-Aldrich^®^, St. Louis, MI, USA), an antioxidant compound analogous to vitamin E, and 3-morpholinosidnonimine (Sin-1, 5 µM) (Sigma-Aldrich^®^, St. Louis, MI, USA), a known oxidant agent, were used as negative and positive control, respectively. Sterile water diluted in HBSS (10%, *v/v*) was used as a control of the treatments. To assess potential protective effect (antioxidant activity) of the samples on ROS production, after 4 h of incubation, all conditions were stressed with Sin-1 (5 µM) and DCF fluorescence was measured for 1 h (every 15 min).

#### 2.4.8. Inflammation Induction

Inflammation was induced in differentiated co-cultures of Caco-2:HT29-MTX with TNF-α (BioLegende^®^, San Diego, CA, USA). The objective was to cause a reduction of at least 20–25% in the transepithelial electrical resistance (TEER) of the cell monolayer to be considered inflamed [37], but without causing destruction of the barrier. Preliminary tests were carried out, using different TNF-α concentrations (10–50 ng/mL) and contact times (4–48 h) on the basolateral and/or apical side. Based on the results only 50 ng/mL of TNF-α for 48 h on the basolateral side was the condition chosen to induce inflammation in the cell monolayers. Co-cultures in MEM were used as the negative control.

##### IL-8 Quantification

After induction of inflammation, basolateral medium was collected and stored at −20 °C for IL-8 quantification using a LEGEND MAX™ Human IL-8 ELISA Kit (Bio-Legend^®^, Inc., San Diego, CA, USA), according to the manufacturer’s instructions. The concentration of human IL-8 was obtained from the interpolation of the absorbance readings (450–570 nm) of the samples using a standard calibration curve.

#### 2.4.9. Effect of TTI on Barrier Integrity and Paracellular Permeability in Inflamed Co-Cultures

The evaluation of the effect of TTI on the barrier integrity and permeability of Lucifer yellow through Caco-2:HT29-MTX monolayers was evaluated in two ways: (1) during the 48 h of inflammation induction, the co-cultures were simultaneously in contact with TTI diluted in culture medium (10%, *v*/*v*), at a final concentration of 1.0 mg/mL; (2) after inducing inflammation for 48 h, the co-cultures were placed in contact with TTI diluted in culture medium (10%, *v*/*v*), at a final concentration of 1.0 mg/mL, for 24 h. In both situations, sterile water diluted in MEM (10%, *v*/*v*) was used as a control of the treatments. Barrier integrity was monitored by measuring TEER and LY permeability.

##### Transepithelial Electrical Resistance (TEER)

The integrity of Caco-2:HT29-MTX cell monolayers during and after inflammation induction was evaluated. The monolayers were left at room temperature to equilibrate for 10 min, and the transepithelial electrical resistance (TEER) was measured using a Millicell^®^ ERS-2 ohmmeter (Merck Millipore, Billerica, MA, USA) with STX electrodes. TEER values were calculated in Ω.cm^2^ by subtracting the TEER of cell-free inserts from the TEER of inserts with cells and multiplying the resulting TEER value by the membrane surface area (1.12 cm^2^).

##### Lucifer Yellow (LY) Permeability

The paracellular transport of Lucifer Yellow through the Caco-2:HT29-MTX monolayers after the inflammation induction was evaluated. The cell culture medium was removed and 1.5 mL of transport medium (HBSS) was added to the basolateral side. On the apical side, a solution (0.5 mL) of 50 µM LY (Sigma-Aldrich^®^, St. Louis, MI, USA) was added and the co-cultures were incubated at 37 °C for 4 h. Samples of 100 µL were collected from the basolateral side every hour (1 h, 2 h, 3 h, and 4 h) to quantify the permeated LY, being replaced by new HBSS (100 µL).

LY fluorescence was measured (λex = 428 nm, λem = 540 nm) using a BioTeK^®^ Synergy H1 microplate reader (Winnoski, VT, USA). TEER was measured at the beginning and at the end of the experiment to monitor possible changes in the barrier integrity. LY concentration was obtained after interpolation of the fluorescence intensity in the calibration curve, prepared using LY solutions from 6.25 µM to 0.0061 µM, in HBSS.

The apparent permeability (Papp) coefficient, in cm/s, was calculated according to the equation: Papp = dQ/dt × V/(A × C0), where dQ/dt is the permeability rate (µM/s), which corresponds to the slope of the cumulative increase in LY concentration in the basolateral chamber over time; V is the volume of the basolateral chamber (1.5 mL), A is the surface area of the insert (1.12 cm^2^), and C0 is the initial concentration of LY in the apical compartment (50 µM).

### 2.5. Preclinical Study

#### 2.5.1. Animals and Study Ethics

Male Wistar rats (*Rattus norvegicus*) with obesity (409 ± 73 g, n = 15) were obtained from the bioterium of Potiguar University (UnP), where the in vivo assays were performed. To induce obesity, newly weaned rats (21 days after birth) were randomly assigned to collective cages (3 animals per cage) and received a high glycemic index and high glycemic load (HGLI) diet for a period of 17 weeks. Obesity was confirmed based on zoometric status (eutrophy = Lee index < 0.3; obesity = Lee index ≥ 0.3) [38]. After confirmation of obesity, the animals were submitted to treatments for ten days, followed by the intestinal permeability test.

During the entire study period, the animals were kept under standard conditions of light (12 h light/dark), temperature (23–25 °C), and humidity (50–55%), and received water and food ad libitum.

The project was submitted and approved by the Ethics Committee on Animal Use of Potiguar University (CEUA-UnP)-protocol nº 019/2017. All experiments were carried out in accordance with the ARRIVE guidelines (Animal Research: Reporting of In Vivo Experiments) [39].

#### 2.5.2. Diets

The diets used in the experiment were:

(A) Standard diet Labina^®^, considered nutritionally adequate for age, commercially obtained (Presence^®^, Paulínia, São Paulo, Brazil);
(B) High glycemic index and high glycemic load (HGLI) diet, described by Luz et al. [40] as inducing obesity and epithelial damage in the intestinal barrier;
(C) Low carbohydrate diet RH195172, commercially obtained (Rhoster^®^, São Paulo, Brazil).

The HGLI diet used to induce obesity, with a high glycemic index (77.6) and high glycemic load (38.8), consisted of the standard Labina^®^ diet, condensed milk, and refined sugar (4.5:4.5: 1 *w/w/w*) and was produced following the methodological procedure described by Luz et al. [40]: to produce 100 g of the HGLI diet, 45.2 g of Labina^®^ diet ground in a food processor, 45.2 mL of condensed milk, and 9.6 g of refined sugar were used having been homogenized manually. Then, the feed was molded into a cylindrical shape and baked in a preheated oven at 180 °C for about 40 min.

#### 2.5.3. Experimental Design

**Figure 1:**
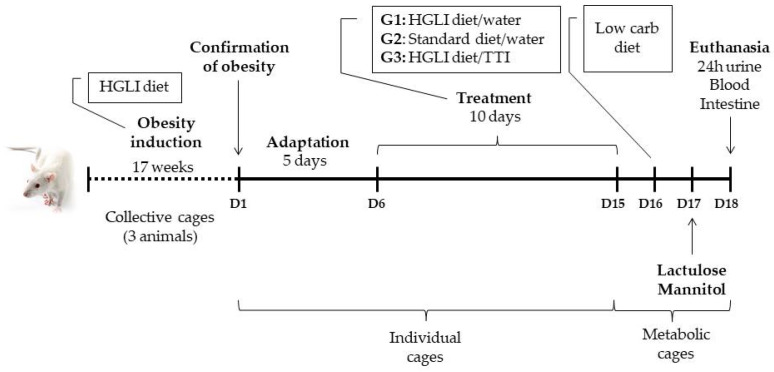
Timeline with the main events of the experimental design of the study with adult male Wistar rats (n = 15). The beginning of the timeline occurred shortly after weaning (21 days after birth). Obesity was previously induced with a high glycemic index and high glycemic load (HGLI) diet. Then, the animals were divided into three groups (n = 5) and subjected to adaptation in individual cages. Thirteen days of experiment followed: ten of treatment and three for the intestinal permeability test. On the 13th day of the experiment, the animals were euthanized for blood and small intestine collection.

After confirmation of obesity, the animals were allocated to individual cages identified and randomly assigned to the following groups (Figure 1):

(1) Group with HGLI diet and no treatment (n = 5), composed of animals with obesity induced by HGLI diet that, during the experimental period, continued to receive this diet and were not treated, receiving only 1 mL of water by gavage;
(2) Group treated with a nutritionally adequate diet (n = 5), composed of animals with obesity induced by the HGLI diet that, during the experiment, received the standard Labina^®^ diet + 1 mL of water by gavage;
(3) Group with HGLI diet and treated with TTI (n = 5), composed of animals with obesity induced by HGLI diet that, during the experiment, continued to receive this diet and were treated with 1 mL of TTI by gavage at a concentration of 25 mg/kg of body weight [27,41].

Before the treatments, the animals underwent five days of adaptation to establish the experimental conditions, when they were submitted to all the daily procedures that occurred during the experiment. After this period, the groups were submitted to the treatments listed above for ten days. Before gavage, the rats were fasted for 8 h.

On the 10th day of treatment, the animals were transferred to metabolic cages, where they remained for three days, two days for adaptation and the third for the intestinal permeability test. For this test, from the 11th day of the experiment, the animals began to receive a low-carbohydrate diet, so that the sugars in the diet did not influence the results. On the 12th day of the experiment, after an 8-h fast, 2.0 mL of a solution containing lactulose (200 mg/mL) and mannitol (50 mg/mL) were administered by gavage. Then, the urine produced during the 24 h after the sugars were offered was collected in the metabolic cage flasks, which already contained 0.25 mL of the antibiotic chlorhexidine (40 mg/mL). At the end of 24 h, the volume of urine of each animal was measured and centrifuged (500× *g*/10 min), and 2.0 mL was removed and stored at −80 °C until the moment of intestinal permeability analysis.

After urine collection, the animals were fasted for 8 h and then anesthetized with 250 mg of tiletamine hydrochloride and 250 mg of zolazepam hydrochloride. Blood (2 mL) was collected by cardiac puncture and the animals were euthanized, with subsequent collection of the small intestine. The intestine was immediately washed with 0.9% saline solution and representative parts of the organ (duodenum, jejunum, and ileum) were separated for histopathological, histomorphometric, and tissue cytokine analyses. Anesthesia, euthanasia, blood, and intestine collections were performed by specialized veterinarians.

#### 2.5.4. Evaluation of Hematological, Biochemical and Inflammatory Parameters

Hematological (hemoglobin, hematocrit, total leukocyte count, and platelet count) and biochemical parameters (fasting blood glucose, total cholesterol and its fractions, glutamic-oxalacetic transaminase (GOT), glutamic-pyruvic transaminase (GPT), gamma glutamyltransferase (GGT), alkaline phosphatase, urea, creatinine, total proteins, and albumin) were evaluated. The measurements were performed using the colorimetric-enzymatic method in automated equipment (Labtest^®^, Natal, RN, Brazil).

The blood destined for the evaluation of plasmatic TNF-α and IL-6 was centrifuged (500× *g*/10 min at 4 °C) to separate the plasma and the amount of TNF-α and IL-6 were quantified using the Quantikine Rat TNF-α Immunoassay and Quantikine Rat IL-6 Immunoassay kits (R&D Systems, São Paulo, Brazil), following the manufacturer’s instructions. All hematological, biochemical, and inflammatory measurements were performed by an evaluator who was blinded to the type of treatment received by each animal.

As reference values for normality, hematological, biochemical, and inflammatory parameters obtained from adults, male, eutrophic (320–380 g), and healthy Wistar rats acclimatized under the same conditions as in this study and fed a standard diet (Labina^®^) were considered. Such reference values, except for cytokines, were also considered in other studies carried out in the same bioterium [42,43] (Table 1).

#### 2.5.5. TNF-α and IL-6 in the Small Intestine

The small intestine of Wistar rats was removed after a longitudinal cut made from the base of the abdomen to the sternum, with the aid of scissors, exposing the entire abdominal and thoracic cavity. Representative fragments obtained by uniform, systematic, and random sampling of the organ (duodenum, jejunum, and ileum) were frozen in liquid nitrogen and kept at −80 °C until the tests. Measurements were made on supernatants (100 µL) of tissue homogenates, obtained by homogenization with PBS (pH 7.4), at a ratio of 1:5, followed by centrifugation at 4 °C for 10 min. The quantification of cytokines was performed by sandwich ELISA, using kits for detection of mouse TNF-α and IL-6 (R&D Systems, Minneapolis, MN, USA), according to the manufacturer’s instructions.

#### 2.5.6. Histopathology and Histomorphometry of the Small Intestine

For histopathology and histomorphometry, representative fragments of the small intestine of the rats, obtained by uniform, systematic, and random sampling of the organ (duodenum, jejunum, and ileum), were fixed in 4% formalin and dehydrated in an increasing alcoholic series (70%, 80%, and 90%), diaphanized in xylene, impregnated, and embedded in histological paraffin. The blocks were sectioned with a microtome (Leica RM2235, Buffalo Grove, IL, USA), obtaining thin slices of paraffin (5 μm) presenting the longitudinal and cross sections of the small intestine. The sections were placed on a microscope glass slide, deparaffinized, rehydrated, and stained with hematoxylin and eosin, covered with a microscope coverslip and analyzed (3 slides with 3 sections each).

The diagnostic reading of the slides was performed blindly by a pathologist. The histopathological evaluation of each specimen was performed using a B-800 microscope (Optika^®^ Microscopes Italy, Ponteranica, Italy). The section images were obtained using a C-P6 digital camera (Optika^®^ Microscopes Italy, Ponteranica, Italy), with a 400× total magnification (Objective Lens 40×). Data were entered into specific forms.

A semi-quantitative evaluation of the main histopathological findings was performed, in which the percentages of intact villi, ulcerated villi, necrotic villi, presence of intestinal glands, and goblet cells were observed. Scores from 0 to 4 were assigned (0: absence; 1: 0 to 25%; 2: 25 to 50%; 3: 50 to 75%; 4: above 75%), according to an adaptation of Silva et al.’s [44] methodology. In addition, the number of inflammatory foci was also evaluated, assigning scores from 0 to 3 (0: absence; 1: 1 to 3 foci; 2: 4 to 6 foci; 3: > 6 foci).

For the histomorphometric analysis, the slides were photographed in the objective with 100× total magnification (Objective Lens 10×) with a C-P6 digital camera (Optika^®^ Microscopes Italy, Ponteranica, Italy), obtaining a panoramic view of the entire specimen. The thickness of the specimens was measured using Proview software (Optika^®^ Microscopes Italy, Ponteranica, Italy). For measurement, three equidistant fixed points (L1, L2, and L3) were established for measuring the thickness of the mucosa and submucosa and the total thickness of the intestinal wall. Thickness was measured with a histological ruler and expressed in µm, obtaining the mean of these measurements for each case analyzed, and then the mean of each group was obtained.

As a reference standard of normality, the morphological aspects of representative slides of the small intestine of adult, male, eutrophic (320–380 g), and healthy Wistar rats, acclimatized under the same conditions of this study and fed with a standard Labina^®^ diet were considered [30].

#### 2.5.7. In Vivo Intestinal Permeability Test

The determination of lactulose and mannitol concentrations in the rats’ urine was performed by High Performance Liquid Chromatography (HPLC), according to the protocol described by Lee et al. [45]. For that, 50 μL of urine was diluted with 50 μL of sorbitol solution (internal standard) and completed with 2.9 mL of distilled and deionized water. Then, the samples were shaken, filtered, and centrifuged before being automatically injected (50 μL) into the HPLC system.

The HPLC system for carbohydrate analysis consisted of: GP40 gradient pump, ED40 electrochemical detector, and AS-40 automatic sample injector (Dionex Co.^®^, Sunnyvale, CA, USA), equipped with a CarboPac^®^ MA1 precolumn and CarboPac^®^ MA1 anion exchange column (4 mm × 250 mm) (Dionex Co.^®^, Sunnyvale, CA, USA). The sugar elution was achieved using isocratic eluent (480 mM NaOH) at a flow rate of 0.4 mL/min. The software for integration and quantification of isolated peaks (analytes) in chromatograms was HPLC PeakNet (Dionex Co.^®^, Sunnyvale, CA, USA).

Sugar concentrations in each injection were calibrated in relation to the amount of internal standard (sorbitol) to compensate for possible variations in the amounts injected between runs. In addition, a standard with known amounts of lactulose, mannitol, sorbitol, and melibiose was applied every nine applications for quality control. Standards were purchased from Sigma-Aldrich^®^ (St. Louis, MI, USA).

The percentage of lactulose excretion (%L) was calculated to measure the increase in permeability or damage of the intestinal epithelium; the percentage of mannitol excretion (%M), to measure the intestinal absorption area; and the lactulose/mannitol (L/M) ratio was used to assess the area of absorption, injury, and repair of the functional intestinal barrier [46].

### 2.6. Statistical Analysis

Sample size (n) of the in vivo study was determined using the Cochran model [47], considering a simple and random sampling. The anticipated coefficient of variation of 10% was adopted with probability of error less than 5% and power of 90%, which corresponded to 4.4 animals, that is, 5 animals per group. Thus, a physiologically significant difference in the parameters evaluated was assumed when the treatment promoted an effect greater than or equal to 25%, according to the 3Rs principle (replacement, reduction, and refinement).

For the statistical treatment of the data, they were initially tabulated in Microsoft Excel program (Microsoft^®^). Statistical tests were performed using GraphPad Prism software, version 5.0 (GraphPad Software, San Diego, CA, USA).

The normality of the distribution was evaluated by the Shapiro–Wilk test to define the best tests to be used for the analysis of numerical data. In the cell study, differences between groups were tested using one-way or two-way ANOVA tests with Bonferroni post-hoc test. In the in vivo study, for parametric variables the one-way ANOVA test with Tukey’s post-hoc test was used. For variables with non-parametric distribution, the Kruskal–Wallis test with Dunn’s post-hoc test was used. Results were considered significant when *p* < 0.05.

## 3. Results

### 3.1. TTI Isolation, Interaction with LPS, and Inhibitory Activity against HNE

**Figure 2:**
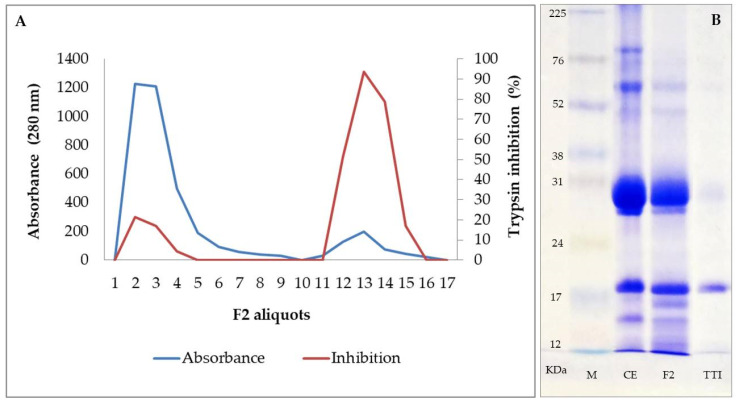
Characterization of the trypsin inhibitor isolated from tamarind seeds. (**A**) Trypsin-Sepharose CNBr 4B affinity chromatography of fraction 2 (F2). A measure of 5 mL of F2 obtained from the crude protein extract of tamarind seeds were applied. The blue line indicates the absorbance at 280 nm of the 3 mL aliquots eluted with 5 mM HCl at a flow rate of 0.5 mL/min; the red line indicates the percentage of antitrypsin activity of the proteins in 100 µL of each eluted aliquot, evaluated using 1.25 mM N-benzoyl-DL-arginine-p-nitroanilide (BApNA) as substrate. (**B**) 12.5% SDS-PAGE showing the electrophoretic profiles of the crude extract obtained from tamarind seeds, of the F2 and of the isolated inhibitor. CE: crude extract; F2: fraction 2 obtained by fractionation with 30–60% of ammonium sulfate; M: marker (GERPN756E, Amersham™ ECL™ Rainbow™ Marker-Full range); TTI: trypsin inhibitor isolated from tamarind seeds.

**Figure 3:**
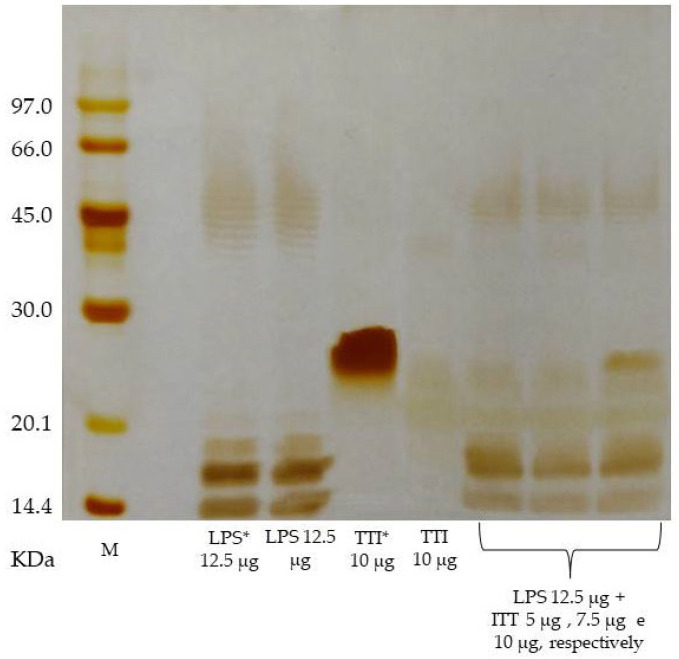
A 12.5% SDS-PAGE, showing the profiles of TTI, LPS, and the product of the incubation of both at 37 °C. The lipopolysaccharide used was obtained from *Escherichia coli* O55:B5 (Sigma-Aldrich^®^). The gel was stained with silver [34]. LPS: lipopolysaccharide; M: marker; TTI: trypsin inhibitor isolated from tamarind seed. (*) Aliquots applied to the gel in the same amounts used in the assay, but without incubation.

Protein fraction 2 (F2), saturated with ammonium sulfate in the range of 30–60%, obtained from the protein extract of tamarind seed flour, showed a high inhibitory activity for trypsin, being, therefore, the fraction submitted to the affinity chromatography for inhibitor isolation. Proteins retained in the Trypsin-Sepharose CNBr 4B column affinity chromatography, eluted with 5 mM HCl at a flow of 0.5 mL/min, showed 97% inhibition of trypsin (771 IU/mg) (Figure 2A). Isolation of the inhibitor was confirmed by 12.5% SDS-PAGE, demonstrating the presence of a major protein band with a molecular mass of approximately 20 kDa (Figure 2B). TTI interaction with LPS was evaluated by SDS PAGE and it was demonstrated that there was no interaction (Figure 3). The inhibition of human neutrophil elastase (HNE) by TTI was assessed and it was found that TTI (30 µg) strongly inhibited HNE (93%).

### 3.2. In Vitro Studies

#### 3.2.1. Cell Metabolic Activity

**Figure 4:**
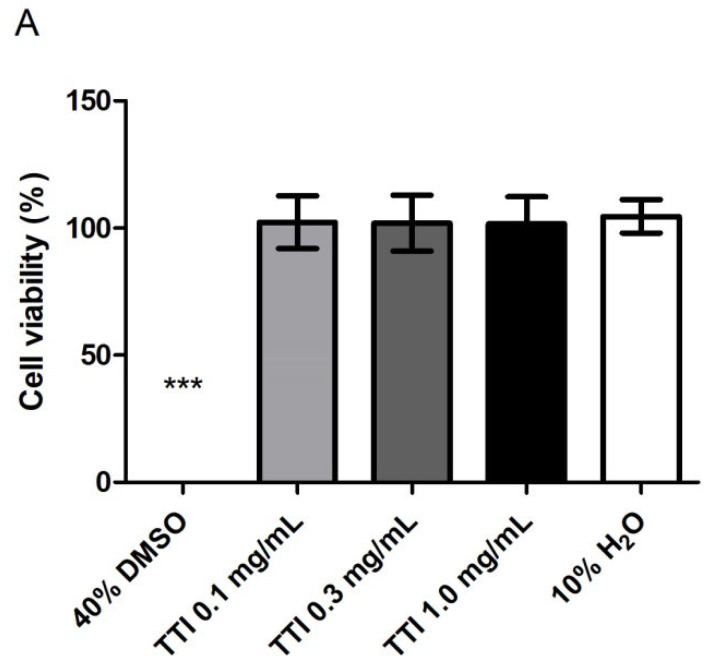

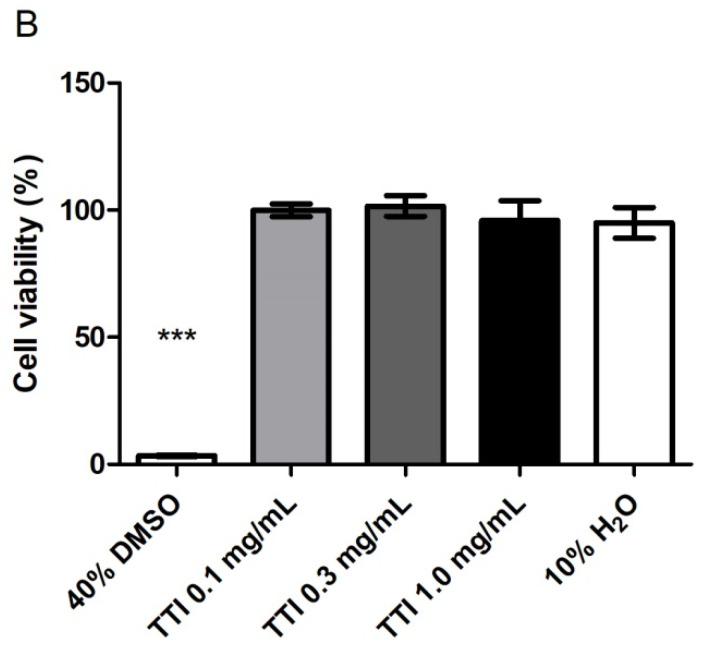
(**A**) Metabolic activity/cell viability of semi-confluent Caco-2 cell cultures after 4 h in contact with the trypsin inhibitor isolated from tamarind seeds (TTI), at different concentrations. (**B**) Metabolic activity/cell viability of differentiated co-cultures of Caco-2:HT29-MTX cells after 24 h in contact with TTI. Cultures in 40% (*v*/*v*) DMSO were used as a negative control and in 10% (v/v) H_2_O as a control of the treatments. Cell viability (%) was calculated based on the fluorescence of the treated cultures, compared to the fluorescence of cultures in MEM (positive control, 100% viability). (**A**,**B**) are the results of two independent assays, with four replicates each. *** *p* < 0.001, compared to the other groups (one-way ANOVA test with Bonferroni post-hoc test). DMSO: dimethylsulfoxide; H_2_O: sterile water.

TTI did not compromise the cell viability of semi-confluent Caco-2 cell cultures after 4 h of contact (Figure 4A), nor after 24 h of contact with co-cultures of Caco-2:HT29-MTX cells differentiated for 21 days (Figure 4B).

#### 3.2.2. Intracellular Production of Reactive Oxygen Species (ROS)

**Figure 5:**
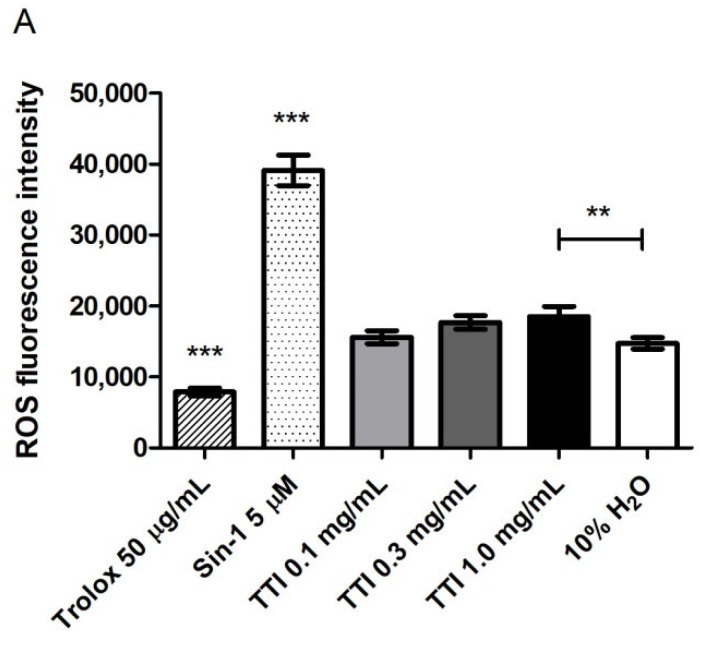

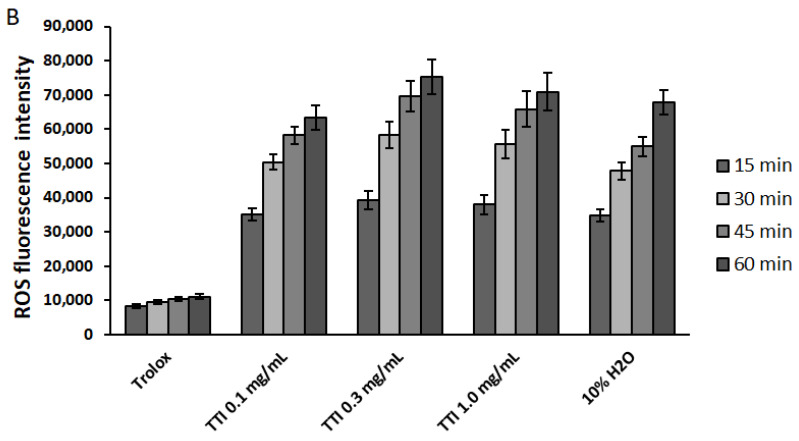
Effect of TTI on basal level of ROS at different concentrations (**A**) after incubation for 4 h with differentiated co-cultures of Caco-2:HT29-MTX cells and (**B**) after additional stress with Sin-1 5 µM, to evaluate the anti-inflammatory potential of the samples. Trolox (50 µg/mL) and Sin-1 (5 µM) were used as negative and positive controls, respectively. H_2_O diluted in HBSS (10% *v*/*v*) was used as a control of the treatments. ** *p* < 0.01; *** *p* < 0.001, compared to the other groups (one-way ANOVA test with Bonferroni post-hoc test). H_2_O: sterile water; Sin-1: 3-morpholinosidnonimine; Trolox: 6-hydroxy-2,5,7,8-tetramethylchroman-2-carboxylic acid.

The maximum TTI concentration tested (1.0 mg/mL), after 4 h in contact with co-cultures of differentiated Caco-2:HT29-MTX cells, led to a higher production of ROS, compared to the untreated control (10% *v*/*v* H_2_O), but this production was significantly lower than that caused by Sin-1, the oxidant used as a positive control (Figure 5A). After the stress induction with Sin-1 in the co-cultures, TTI did not prevent the additional production of ROS by the cells, which increased over time and was as high as in the not treated cells (10% *v*/*v* H_2_O) and significantly higher when compared to the production in co-cultures treated with the antioxidant Trolox (Figure 5B).

#### 3.2.3. Inflammation Induction in Cell Co-Cultures

The induction of inflammation in Caco-2:HT29-MTX co-cultures was confirmed, considering the following parameters: transepithelial electrical resistance (TEER), Lucifer Yellow (LY) permeability through cell monolayers and quantification of IL-8 in the basolateral medium of these co-cultures (Figure S1).

#### 3.2.4. Effect of TTI on Transepithelial Electrical Resistance (TEER) in Inflamed Co-Cultures

**Figure 6:**
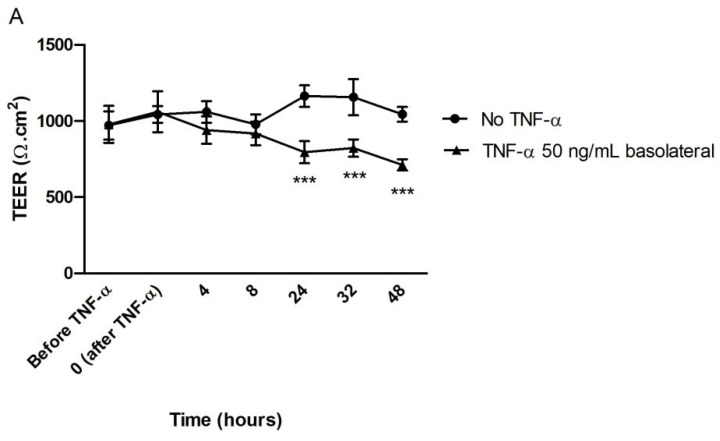

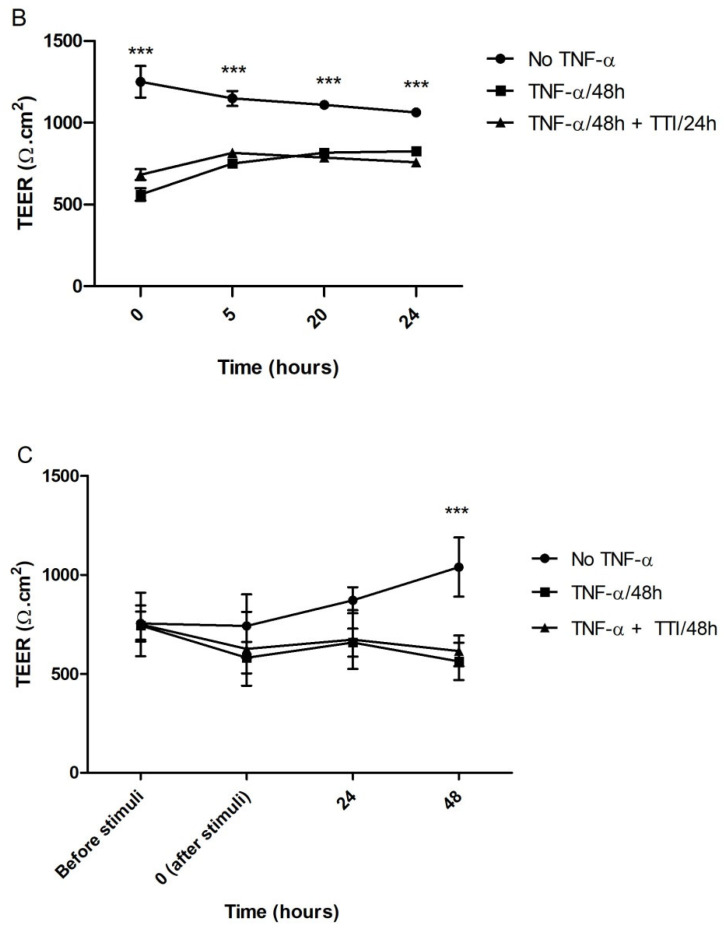
Transepithelial electrical resistance (TEER) of inflamed monolayers of Caco-2:HT29-MTX cells. (**A**) During induction of inflammation with TNF-α 50 ng/mL on the basolateral side, for 48 h. (**B**) During treatment with TTI 1.0 mg/mL for 24 h, on previously inflamed co-cultures. (**C**) During treatment with TTI 1.0 mg/mL simultaneous to inflammation with TNF-α 50 ng/mL on the basolateral side, for 48 h. *** *p* < 0.001, in relation to the other groups (2-way ANOVA test with Bonferroni post-hoc test). TNF-α: tumor necrosis factor alpha; TTI: trypsin inhibitor isolated from tamarind seeds.

The results of TEER measurements over the 48 h of induction of inflammation with TNF-α (50 ng/mL, on the basolateral side) demonstrate that, after 24 h in contact with the cytokine, the differentiated monolayers of Caco-2:HT29-MTX already showed a significant reduction (−18%) in the barrier integrity when compared to control monolayers, and this reduction was greater after 48 h (−27%) (Figure 6A). However, TTI (1.0 mg/mL) did not help restoring this integrity neither when the treatment occurred after the inflammation (Figure 6B) nor when applied simultaneously to the inflammatory stimulus (Figure 6C).

#### 3.2.5. Effect of TTI on Permeability in Inflamed Co-Cultures

**Figure 7:**
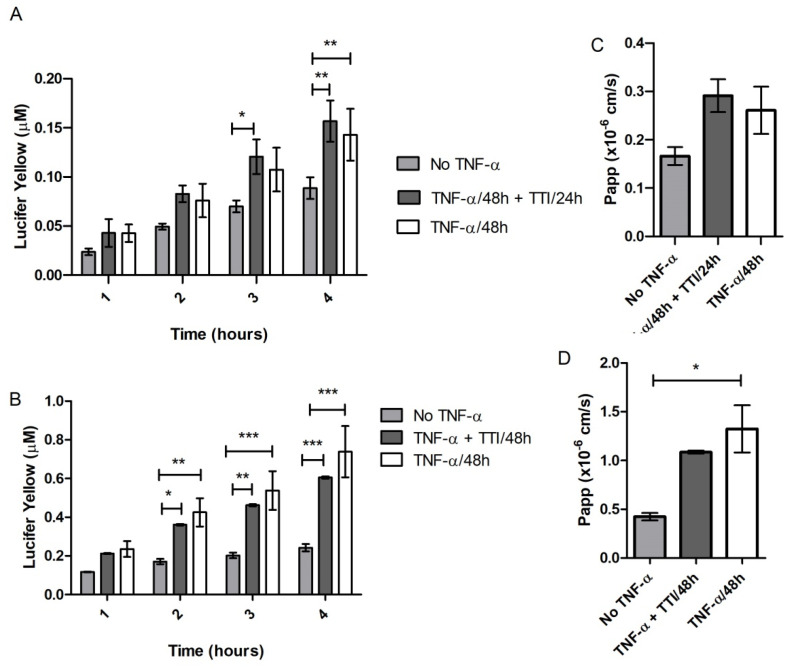
Concentration of Lucifer Yellow (LY) permeated to the basolateral side over 4 h (**A**,**B**) and apparent permeability (**C**,**D**) in co-cultures of Caco-2:HT29-MTX cells stimulated with TNF-α 50 ng/mL on the basolateral side for 48 h. Treatment with TTI 1.0 mg/mL was performed (**A**,**C**) after inflammation for 24 h or (**B**,**D**) during inflammation for 48 h. * *p* < 0.05; ** *p* < 0.01; *** *p* < 0.001; in (**A**,**B**): two-way ANOVA test with Bonferroni post-hoc test; in (**C**,**D**): one-way ANOVA test with Bonferroni post-hoc test. Paap: apparent permeability; TTI: trypsin inhibitor isolated from tamarind seeds.

TTI treatments did not reduce Lucifer Yellow permeation through cell monolayers; the basolateral LY concentrations over time and the respective apparent permeability between TTI-treated inflamed co-cultures (after or during stimulation) and untreated inflamed co-cultures (no TNF-α) are similar (Figure 7).

### 3.3. Preclinical Study

#### 3.3.1. Hematological and Biochemical Parameters

The results of the hematological and biochemical parameters of the animals after treatment with TTI did not differ significantly from the group of untreated animals, except for total cholesterol and HDL-c and LDL-c fractions, which were significantly lower in the group of animals treated with TTI (Table 2). Some concentrations (hemoglobin, total leukocyte count, platelets, and fasting blood glucose), even though they did not differ from the values of the untreated group, were significantly lower than those presented by animals treated with the nutritionally adequate diet.

#### 3.3.2. Plasma TNF-α and IL-6

**Figure 8:**
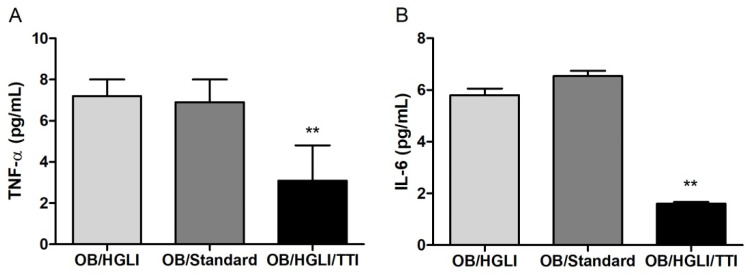
Plasma concentrations of inflammatory cytokines in Wistar rats with obesity induced by HGLI diet, after ten days of experiment. (**A**) Plasma TNF-α in animals in the groups: without treatment (OB/HGLI), treated with a nutritionally adequate diet (standard diet Labina^®^) (OB/Standard) and treated with the trypsin inhibitor isolated from tamarind seeds (TTI) (OB/HGLI/TTI). ** *p* < 0.01 (one-way ANOVA test with Tukey’s post-hoc test); (**B**) plasma IL-6 in animals in the groups: without treatment (OB/HGLI), treated with a nutritionally adequate diet (OB/Standard) and treated with the trypsin inhibitor isolated from tamarind seeds (TTI) (OB/HGLI/TTI). ** *p* < 0.01 (Kruskal–Wallis test with Dunn’s post-hoc test). HGLI: high glycemic index and high glycemic load diet; IL-6: interleukin 6; OB: obesity; TNF-α: tumor necrosis factor alpha.

Regarding plasma TNF-α, the mean concentration observed in the group of animals treated with TTI (3.7 ± 0.9 pg/mL) was significantly lower when compared to the untreated animals (6.8 ± 1.4 pg/mL) and treated only with the nutritionally adequate diet (standard diet Labina^®^) during the experiment (6.6 ± 1.4 pg/mL) (*p* = 0.003) (Figure 8A).

In relation to IL-6 values, the group of animals treated with TTI had a mean of 1.6 ± 0.2 pg/mL, significantly lower (*p* = 0.004) compared to the means of the untreated animals (5.8 ± 0.6 pg/mL) and treated with a nutritionally adequate diet (6.5 ± 0.5 pg/mL) (Figure 8B).

#### 3.3.3. TNF-α and IL-6 in the Small Intestine

**Figure 9:**
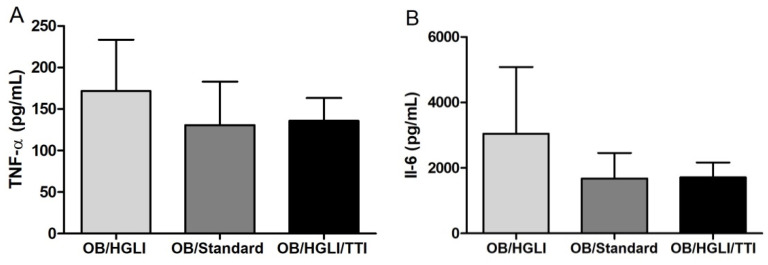
Inflammatory cytokine concentrations in small intestine homogenates of Wistar rats with obesity induced by HGLI diet, after ten days of the experiment. (**A**) Mean concentration of TNF-α in small intestine homogenates of animals in the groups: without treatment (OB/HGLI), treated with a nutritionally adequate diet (standard diet Labina^®^) (OB/Standard) and treated with the trypsin inhibitor isolated from tamarind seeds (TTI) (OB/HGLI/TTI). (**B**) Mean concentration of IL-6 in small intestine the homogenates of animals in the groups: without treatment (OB/HGLI), treated with a nutritionally adequate diet (OB/Standard) and treated with the trypsin inhibitor isolated from tamarind seeds (TTI) (OB/HGLI/TTI). *p* > 0.05 (Kruskal–Wallis test with Dunn’s post-hoc test). HGLI: high glycemic index and high glycemic load diet; IL-6: interleukin 6; OB: obesity; TNF-α: tumor necrosis factor alpha.

The results of the measurements of inflammatory cytokines in the animals’ small intestine homogenates did not show significant differences between the groups (*p* = 0.902 and *p* = 0.944 for TNF-α and IL-6, respectively) (Figure 9).

#### 3.3.4. Histopathology and Histomorphometry

**Figure 10:**
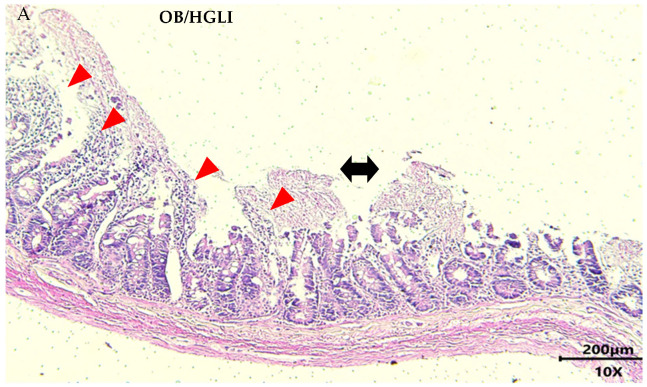

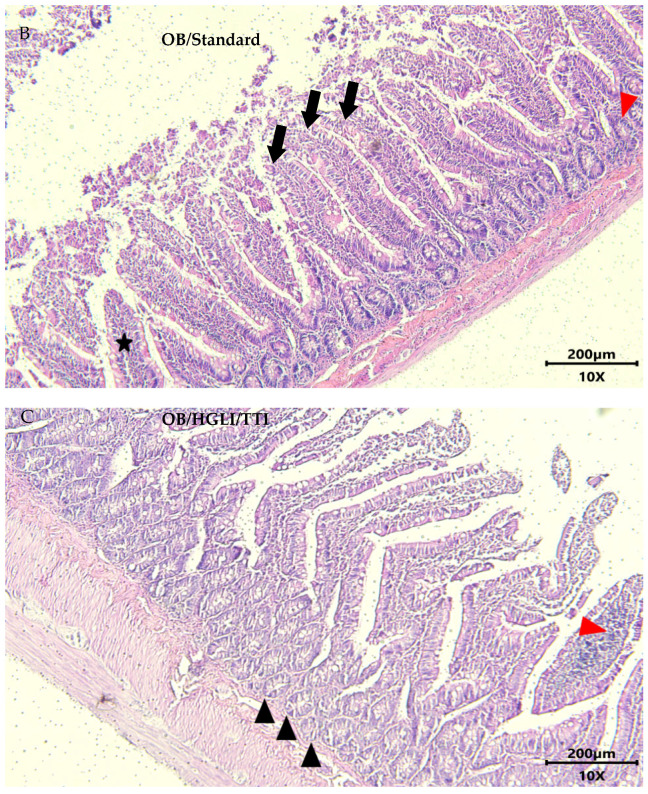
Histological slides representative of the small intestine of Wistar rats with obesity induced by HGLI diet, after ten days of experiment, stained with hematoxylin-eosin. (**A**) Group without treatment (OB/HGLI). (**B**) Group treated with a nutritionally adequate diet (standard diet Labina^®^) (OB/Standard). (**C**) Group treated with the trypsin inhibitor isolated from tamarind seeds (TTI) (OB/HGLI/TTI). Scale bar: 200 μm. Objective Lens: 10×. Total Magnification: 100×. Signals point to intact villus (star), ulcerated villus (arrow), areas of mononuclear inflammatory infiltrate (red arrowhead), necrotic villus (double arrow), and intact intestinal gland (black arrowhead). OB: obesity; HGLI: high glycemic index and high glycemic load diet.

All rats in the untreated group had moderate to severe intestinal inflammation. Animals treated with a nutritionally adequate diet were similar, with moderate to severe chronic enteritis, and only one rat presented a mild degree of enteritis. In the group treated with TTI, all animals had mild chronic enteritis (Figure 10).

**Figure 11:**
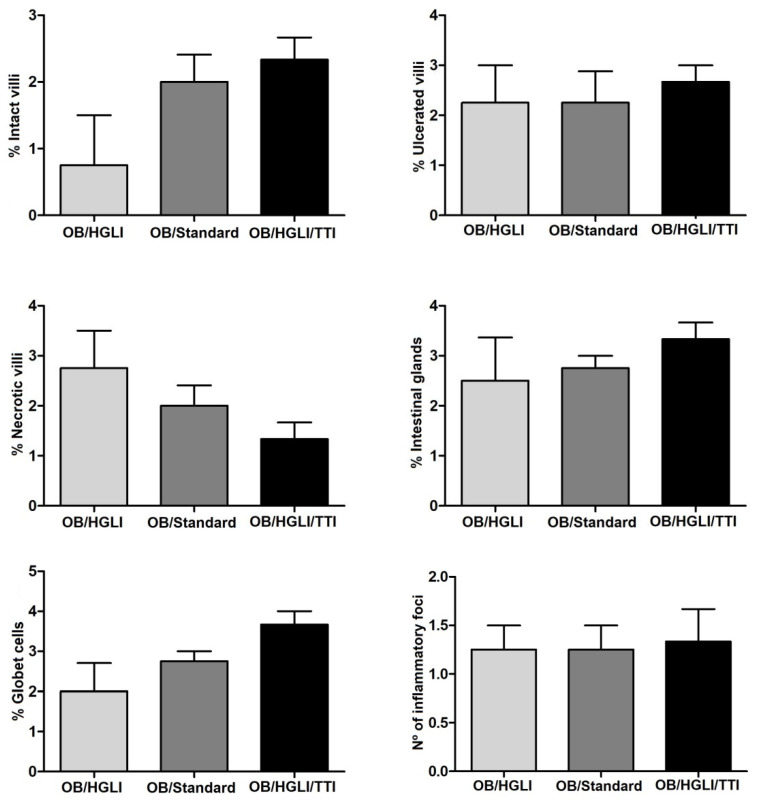
Semiquantitative histopathological analysis of intestinal parameters evaluated in Wistar rats with obesity induced by HGLI diet, after ten days of experiment. OB/HGLI: group without treatment, OB/Standard: group treated with a nutritionally adequate diet (standard diet Labina^®^) and OB/HGLI/TTI: group treated with the trypsin inhibitor isolated from tamarind seeds (TTI). The scores related to the percentage of intact villi, villous necrosis, presence of intestinal glands, and the amount of inflammatory foci that had a nonparametric distribution, so the Kruskal–Wallis test with Dunn’s post-hoc test was used to verify differences between pairs. The other parameters presented a parametric distribution, being performed by one-way ANOVA test with Tukey’s post-hoc test. OB: obesity; HGLI: high glycemic index and high glycemic load diet.

**Figure 12:**
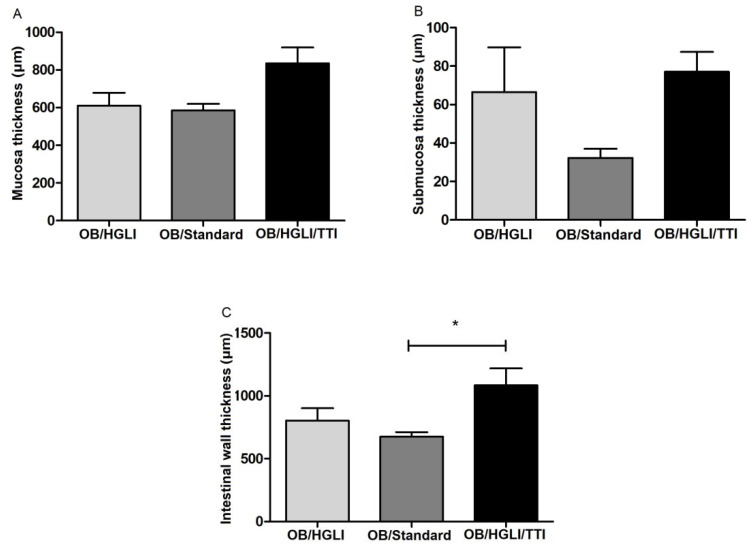
Histomorphometric analysis of intestinal parameters evaluated in Wistar rats with obesity induced by HGLI diet, after ten days of experiment. (**A**) Mucosa thickness, (**B**) submucosa thickness, and (**C**) intestinal wall thickness. OB/HGLI: group without treatment, OB/Standard: group treated with a nutritionally adequate diet (standard diet Labina^®^), and OB/HGLI/TTI: group treated with the trypsin inhibitor isolated from tamarind seeds (TTI). * *p* < 0.05 (one-way ANOVA test with Tukey’s post-hoc test). OB: obesity; HGLI: high glycemic index and high glycemic load diet.

Although we did not observe significant differences in most of the semiquantitative and histomorphometric analyses (Figure 11 and Figure 12), the detailed histopathological description and the values of total intestinal wall thickness (Figure 12) of the small intestines showed some differences between the groups.

In relation to the animals in the untreated group, there was a greater heterogeneity of intestinal findings, as some animals showed large extensions of absence of villi, while others showed greater preservation. There were also ulcerated areas and inflammatory infiltrate in all slides analyzed, in addition to areas of necrosis. Regarding the preservation of intestinal glands and goblet cells, the findings were also heterogeneous within the group (Figure 10A). In the histomorphometry, the animals of this group presented a greater thickness of the intestinal wall, a greater edema in the mucosa and lesser atrophy of the submucosa, in relation to the group treated with the nutritionally adequate diet.

Animals in the group treated with the nutritionally adequate diet showed some preservation of intestinal villi lined by simple columnar epithelium but had areas of epithelial discontinuity and exposure of underlying connective tissue (ulcers) with mononuclear inflammatory infiltrate. The findings were heterogeneous among the animals regarding the percentage of necrotic villous areas and destruction of intestinal glands and goblet cells in the lamina propria (Figure 10B). In the histomorphometry, the animals of this group exhibited the smallest thickness of the intestinal wall, an increase in the mucosa’s thickness caused by edema, and remarkable atrophy of the submucosa.

The animals in the TTI-treated group showed greater preservation of intestinal villi integrity, despite showing areas of epithelial discontinuity and ulceration with inflammatory infiltrate. There was also a greater scarcity of areas of necrosis and absence of villi. In the lamina propria, there was a greater number of intestinal glands and greater preservation of goblet cells. The muscular layer of the mucosa presented aspects of normality, while the mucosa and serosa layers showed greater thickening (Figure 10C). In the histomorphometry, we observed a greater thickness of the entire intestinal wall, with more edema in the mucosa and less atrophy in the submucosa, compared to animals from the other groups, making it clear that there was greater preservation of the submucosal layer than in the mucosa.

#### 3.3.5. Intestinal Permeability

**Figure 13:**
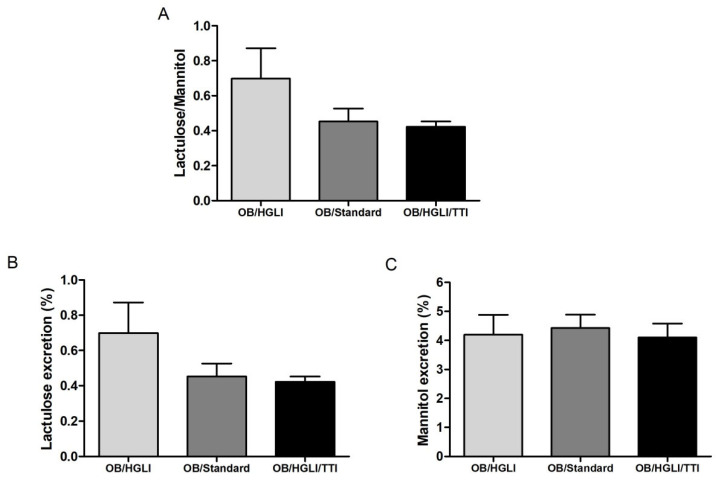
Intestinal permeability in Wistar rats with obesity induced by HGLI diet, after ten days of experiment. OB/HGLI: group without treatment, OB/Standard: group treated with a nutritionally adequate diet (standard diet Labina^®^), and OB/HGLI/TTI: group treated with the trypsin inhibitor isolated from tamarind seeds (TTI). The animals received gavage with 2.0 mL lactulose (200 mg/mL) and mannitol (50 mg/mL) solution, followed by urine collection for 24 h to evaluate the percentages of lactulose and mannitol excretion. (**A**) Mean lactulose/mannitol excretion rates in the urine samples of the animals. (**B**) Mean percentages of lactulose excretion in the urine samples of the animals. (**C**) Mean percentages of mannitol excretion in the urine samples of the animals. *p* > 0.05 (Kruskal–Wallis test with Dunn’s post-hoc test). OB: obesity; HGLI: high glycemic index and high glycemic load diet.

There was no significant difference for the lactulose/mannitol (L/M) excretion ratio (*p* = 0.765) between the animals of the three groups (Figure 13), or for the isolated percentages of excretion of lactulose (*p* = 0.778) and mannitol (*p* = 0.934).

## 4. Discussion

Evidence indicates that the combination of food quantity and quality, together with genetic susceptibility, induces an abnormal activation of innate immune signaling, which contributes to a low-grade chronic inflammatory state, common in many chronic non-communicable diseases (NCDs), such as obesity, diabetes, and cardiovascular disease [48].

The increase in the incidence of NCDs has aroused interest in researching alternatives to the classic forms of treatment, such as trypsin inhibitor proteins, which have shown positive results in the treatment of obesity due to their action on mechanisms related to satiety and inflammation. In addition, trypsin inhibitors improve biochemical parameters related to obesity and metabolic syndrome, being considered promising candidates in the treatment and prevention of chronic diseases [26,27,28,49,50].

In the present study, we isolated and evaluated a tamarind protein identified as trypsin inhibitor isolated from tamarind seeds (TTI). The inhibition profile against trypsin and the molecular mass of the TTI confirmed that it is the same molecule previously isolated by the group, demonstrating the reproducibility and standardization of the isolation method.

Isolated TTI inhibited HNE in vitro, an activity that had already been described for a 14 kDa inhibitor isolated from tamarind seeds [29]. However, in our study, the TTI profile visualized on SDS-PAGE showed a major protein band with a molecular mass of approximately 20 kDa. Therefore, we believe this protein’s HNE inhibition activity is unprecedented. This is an important result, as HNE is involved in the gut recruitment, influx, and infiltration of neutrophils [18]. Furthermore, macrophage infiltration is also linked to HNE activity, so its inhibitors also affect macrophages [51].

Han and Levings [52] highlighted that neutrophils can transiently infiltrate adipose tissue and that the exacerbated production of elastase contributes to inflammation in this tissue. In addition, increased HNE activity was detected in the serum of obese humans, and HNE-deficient mice were protected against diet-induced obesity and insulin resistance.

In this study, the isolated protein was also tested in vitro for interaction with LPS for inferences about a possible antibacterial effect. However, SDS-PAGE showed that TTI did not interact with *E. coli* LPS at the concentrations tested. Additionally to antibacterial activity, antioxidant activity is displayed by many bioactive compounds and is associated with the recovery of intestinal barrier integrity [53,54,55]. In this study, however, we analyzed the TTI’s ability to prevent ROS production in co-cultures of Caco-2:HT29-MTX cells after stress with Sin-1, and the results pointed to the absence of antioxidant potential of TTI, up to a concentration of 1.0 mg/mL. Furthermore, in tests of oxidative stress, TTI did not show pro-oxidant activity, not inducing ROS production in the cell cultures.

Regarding cytotoxicity tests, TTI did not change the cell viability when tested in contact with semiconfluent cultures of Caco-2 cells for 4 h or with differentiated monolayers of Caco-2:HT29-MTX cells for 24 h. The absence of cytotoxicity of TTI in contact with semiconfluent Caco-2 cells was previously reported by Costa et al. [43], but with the TTI nanoencapsulated in chitosan and whey protein (1:2:2).

The fact that TTI did not change cell viability or Increase ROS production in co-cultures justifies the choice of a concentration of 1.0 mg/mL to be tested in subsequent analyses, which aimed to verify if TTI could prevent damage or restore the integrity of TNF-α-inflamed intestinal cell monolayers. However, the results showed that TTI, under the studied conditions, did not prevent the loss or improved the recovery of the barrier integrity of the cell monolayer from the damage caused by inflammation. We verified this by evaluating both the transepithelial electrical resistance (TEER) of the monolayers and the permeability of Lucifer Yellow, which is a molecule transported through the paracellular pathway.

Despite the results obtained in the study with the inflamed cell cultures, the previous findings of the group in Wistar rats with obesity, that showed a reduction in plasma TNF-α after treatment with TTI, stimulated our investigation regarding the anti-inflammatory activity of this protein and its effects on the intestinal barrier in a preclinical model of diet-induced obesity.

Regarding the safety aspects and harmful effects of the TTI dose used in animals, we observed through the hematological and biochemical parameters, that the treatment with 25 mg/kg/day for ten days did not lead to significant differences in most of the evaluated markers. Obesity was induced by the HGLI diet, which causes changes in most of the parameters evaluated, as observed by Luz et al. [40], who pointed out effects harmful effects of chronic consumption of this diet. However, the results showed that TTI did not worsen the hematological parameters, glycemia, lipid, hepatic, and renal profiles of the animals, corroborating what was observed by Carvalho et al. [27], when they evaluated the effect of TTI in Wistar rats with obesity and metabolic syndrome, also induced by the HGLI diet.

We observed statistically different concentrations between animals treated and not treated with TTI in the parameters of total cholesterol, HDL-c, and LDL-c. The serum LDL-c concentration in the TTI-treated animals was almost half of that seen in the untreated animals, an unprecedented finding. This result is positive, as a lower concentration of this type of cholesterol has been associated with a reduction in morbidity and mortality associated with cardiovascular diseases [56,57].

Compared to the animals treated with the nutritionally adequate diet, in addition to the lipid profile, the rats treated with TTI showed significant differences in the values of hemoglobin, total leukocyte count, platelets, and fasting blood glucose. Observing the hematological parameters, however, the values found in the animals treated with TTI remained within the reference ranges for healthy and eutrophic animals (Table 1).

The lower total leukocyte count observed in the treated animals may be related to a reduction in systemic inflammation because the white blood cell count is a marker of subclinical inflammation, which may be associated with insulin resistance and be used as an indicator of the risk of metabolic syndrome and type 2 diabetes in individuals with obesity [58,59].

Therefore, the lower total leukocyte count may be associated with significantly lower fasting blood glucose in animals treated with TTI, compared to animals that received a nutritionally adequate diet. This aspect is positive since diets with high sugar levels are related to changes in the glycemic profile of individuals [60]. TTI has already demonstrated a significant effect on fasting blood glucose in Wistar rats with obesity induced by the HGLI diet, which had a lower glycemic value than untreated animals [41].

Additionally, isolated or nanoencapsulated TTI was a safe molecule in animals, with no signs of toxicity observed in this or other studies of the group, considering hematological and biochemical parameters related to pancreatic, renal, and hepatic functions [26,27,42,43,61]. In addition, the analyses carried out in these previous studies on morphological aspects of organs (stomach, intestine, pancreas, and liver), which are related to macroscopic and cellular damage due to toxic effects, were within the standards, with the same dose of administration and the same experimental period of this study.

We used the HGLI diet because experimental and epidemiological studies point to adverse effects of excessive sugar-rich foods and beverages, concluding that glycation after high sugar intake may play a central role in developing metabolic disorders. Thus, glycation interferes with the signaling of many cellular pathways, influencing the inflammatory and pro-oxidant state that contributes to tissue damage and organ dysfunction [62].

Inflammation caused by the HGLI diet was studied by Luz et al. [40], who evaluated the inflammatory effect of this diet in Wistar rats and observed that, when consumed for 17 weeks, it led to adipocyte hypertrophy, greater fat deposition in the liver and pancreas, greater gene expression and plasma concentration of TNF-α, alteration, and inflammation in the intestinal epithelium when compared to animals that consumed a nutritionally adequate diet. Thus, according to these authors, the results showed that the HGLI diet generated alterations commonly observed when there is a chronic consumption of foods and drinks rich in fat or sugar.

In our study, this inflammatory state was evidenced by the high plasma concentrations of TNF-α and IL-6 in untreated animals and even in those treated with a nutritionally adequate diet, in which obesity was induced for 17 weeks with HGLI diet. Only animals treated with TTI had significantly lower concentrations of inflammatory cytokines in plasma, with values similar to those of eutrophic and healthy animals (Table 1), evidencing the anti-inflammatory potential of this molecule. As mentioned above, a lower TNF-α concentration in plasma was already observed by Carvalho et al. [27] in animals with obesity and metabolic syndrome treated with 25 mg/kg/day of TTI for ten days. However, compared to untreated animals, the lower IL-6 plasma concentration had not yet been reported as an effect of TTI treatment.

It is important to emphasize that, despite the systemic (plasma) reduction, we did not observe cytokines at the local level, analyzing the homogenate of the small intestine of the animals. We assume that the plasma reduction occurred due to the reduction of TNF-α in adipose tissue, as reported by Carvalho et al. [63]. The significant reduction in plasma TNF-α may also be related to the reduction in leukocytes and, in some way, have contributed to the subtle improvement of tissue aspects in the animals’ intestine, although not enough to improve the permeability of the intestinal barrier.

When evaluating the histopathological description of the small intestine of the rats, all animals had chronic enteritis, presenting many aspects of loss of intestinal wall integrity, such as ulceration, necrosis, and absence of villi, presence of mononuclear inflammatory infiltrate, and submucosa atrophy. However, the animals in the group treated with TTI, despite showing histopathological changes, had a greater number of intact villi, intestinal glands, and goblet cells, and less leukocyte infiltration, in addition to greater preservation of the submucosal layer, which reflected in a greater thickness of the total intestinal wall. These are the findings that most resemble the aspects of normality used as a reference in this study [30].

Lima et al. [30] observed that animals with obesity induced by the HGLI diet and treated for ten days with TTIp showed less ulceration and necrosis of the villi, compared to untreated animals. These data corroborate the results of this study.

Despite the histopathological findings demonstrating the positive effects of the treatment with TTI, we did not observe significant differences between the three groups regarding intestinal permeability, both the isolated percentages of lactulose and mannitol excretion and the lactulose/mannitol (L/M) ratio were statistically similar between the groups, similar to what we observed in the LY permeability test in co-cultures of Caco-2:HT29-MTX cells. These results indicate that TTI probably had another action on the intestinal epithelium, which is not related to changes in tight junction proteins and, consequently, in paracellular permeability.

Nguyen et al. [64] also found no significant differences in the L/M ratio in Wistar rats fed a high-fat diet and treated with an anti-inflammatory molecule. However, unlike our findings, the percentage of mannitol excretion was lower, leading the authors to infer that there was an improvement in intestinal barrier function after treatment.

A recent systematic review of intestinal permeability studies investigated whether obesity, with or without metabolic syndrome, is associated with impaired intestinal barrier function in humans and concluded that there is no definitive evidence on the association between these conditions. Studies that performed the lactulose/mannitol test did not find differences between the groups of eutrophic and obese individuals; a greater permeability of these sugars in obesity was not observed. Some studies with serum zonulin, on the other hand, found a positive association [65]. Therefore, further studies are needed to understand if TTI reduces intestinal permeability in obese animals, using other markers and including a group of eutrophic animals for comparison.

In summary, the reduction of the mononuclear inflammatory infiltrate observed in the intestine of the animals treated with TTI, added to the positive aspects related to the morphology and integrity of the intestinal epithelium, point to the hypothesis that the in vitro anti-elastase activity presented by the TTI could justify or be related to these in vivo results.

Some limitations of this study should be mentioned, such as the contact of the intact TTI (not digested) with cell cultures, and the absence of a control group of eutrophic and healthy animals, to show more clearly the changes induced by the HGLI diet. However, in previous studies [30,40], the small intestine of eutrophic and healthy animals, set in the same conditions of this study, showed no alterations, with good preservation of the evaluated structures, serving as a reference of normality.

Future perspectives in the study of TTI effects on the intestine include the analysis of markers associated with changes in the intestinal barrier, classical models of inflammatory bowel diseases, and collection of the large intestine. Future studies should also assess the antimicrobial activity of TTI and its action on the intestinal microbiota, regardless of the absence of interaction with LPS presented in our study. Considering the lower degree of enteritis in animals treated with TTI, despite the absence of antioxidant activity or improvement in intestinal permeability, it is expected to understand how TTI acted leading to lower plasma concentrations of TNF-α, IL-6, and total leukocytes, how this influenced the tissue aspects of the intestine, and if the inhibition of HNE is related to the results in vivo. Our study showed that TTI represents a promising protein for clinical application and may become an option as a novel ingredient in food or drug development and formulation.

## 5. Conclusions

In conclusion, the trypsin inhibitor isolated from tamarind seeds (TTI), at the concentrations tested in vitro, strongly inhibited HNE, did not interact with bacterial LPS, and did not alter cell viability nor induce oxidative stress in differentiated monolayers of Caco-2:HT29MTX cells. TTI did not show antioxidant properties, nor the ability to prevent damage or restore the barrier integrity of cell monolayers inflamed with TNF-α. In the preclinical study, treating animals with diet-induced obesity with TTI, for ten days, the test molecule did not worsen hematological and biochemical parameters, which showed some changes caused by the high glycemic index and high glycemic load diet used. The treatment resulted in lower plasma concentrations of total leukocytes, inflammatory cytokines, and other obesity-related parameters, such as fasting glucose and LDL-c; it also led to a lower degree of enteritis caused by chronic diet consumption, regardless of the effect on the concentration of these cytokines in the intestine and on intestinal permeability.

## Figures and Tables

**Table 1:** Reference hematological, biochemical, and inflammatory parameters for male, adult, eutrophic, and healthy Wistar rats acclimatized in the bioterium of Potiguar University, Natal, RN, Brazil, 2019.

Parameter (Unit)	Reference Value * [42,43]
Hemoglobin (g/dL)	23.90 (15.70)
Hematocrit (%)	39.80 (4.82)
Total leukocyte count (×10^3^/µL)	6.44 (0.66)
Platelets (×10^5^/µL)	3.41 (0.69)
Fasting blood glucose (mg/dL)	88.80 (17.87)
Total cholesterol (mg/dL)	112.00 (54.00)
HDL-c (mg/dL)	23.40 (4.04)
LDL-c (mg/dL)	22.76 (4.05)
VLDL-c (mg/dL)	20.65 (5.59)
GOT (U/L)	49.20 (11.90)
GPT (U/L)	43.40 (6.11)
GGT (U/L)	33.50 (3.38)
Alkaline phosphatase (U/L)	64.50 (6.59)
Urea (mg/dL)	32.20 (7.08)
Creatinine (mg/dL)	0.80 (0.16)
Total proteins (mg/dL)	6.46 (0.42)
Albumin (mg/dL)	2.30 (0.26)
TNF-α (pg/mL)	3.16 (0.54)
IL-6 (pg/mL)	1.30 (0.16)

* Mean (standard deviation). HDL-c: high-density lipoprotein cholesterol; LDL-c: low-density lipoprotein cholesterol; VLDL-c: very low-density lipoprotein cholesterol. GOT: glutamic-oxalacetic transaminase; GPT: glutamic-pyruvic transaminase; GGT: gamma glutamyltransferase; TNF-α: tumor necrosis factor-alpha; IL-6: interleukin 6.

**Table 2:** Hematological and biochemical parameters of Wistar rats with obesity induced by HGLI diet, after ten days of experiment.

Parameters	OB/HGLI	OB/Standard	OB/HGLI/TTI	*p* Value *
Hemoglobin (g/dL)	13.4 (0.6) ^a^	14.3 (1.3) ^a.b^	12.8 (0.6) ^a.c^	0.029
Hematocrit (%)	39.0 (0.7)	29.8 (13.8)	38.5 (1.4)	0.970
Total leukocyte count (×103/µL)	8.30 (0.40) ^a^	8.64 (0.48) ^a.b^	6.04 (0.39) ^a.c^	0.007
Platelets (×105/µL)	3.93 (0.53) ^a^	4.32 (0.51) ^a.b^	3.02 (0.83) ^a.c^	0.021
Fasting blood glucose (mg/dL)	151 (34.3) ^a^	167.5 (17.5) ^a.b^	107.4 (7.1) ^a.c^	0.015
Total cholesterol (mg/dL)	84.1 (7.6) ^a^	79.4 (12.2) ^a^	58.6 (8.5) ^b^	0.003
HDL-c (mg/dL)	27.7 (2.2) ^a^	34.6 (3.9) ^b^	22.0 (2.2) ^c^	0.000
LDL-c (mg/dL)	40.9 (5.2) ^a^	30.6 (10.7) ^a.b^	22.8 (6.4) ^b^	0.011
VLDL-c (mg/dL)	15.5 (1.6)	14.2 (1.3)	15.8 (1.7)	0.267
GOT (U/L)	45.8 (4.5)	36.2 (7.3)	59.0 (8.6)	0.093
GPT (U/L)	43.6 (3.5)	40.7 (9.2)	46.6 (3.4)	0.339
GGT (U/L)	28.1 (1.9)	27.9 (2.3)	28.7 (1.1)	0.808
Alkaline phosphatase (U/L)	65.1 (5.4)	67.3 (4.8)	62.4 (4.6)	0.321
Urea (mg/dL)	26.1 (2.0)	25.9 (2.3)	27.1 (2.8)	0.684
Creatinine (mg/dL)	0.8 (0.2)	0.8 (0.2)	0.8 (0.2)	0.865
Total proteins (mg/dL)	6.5 (0.4)	6.3 (0.3)	6.7 (0.2)	0.241
Albumin (mg/dL)	3.0 (0.2) ^a^	3.5 (0.2) ^b^	2.8 (0.3) ^a^	0.000

OB/HGLI: No treatment group; OB/Standard: Group treated with a nutritionally adequate diet (standard diet Labina^®^); OB/HGLI/TTI: Group treated with trypsin inhibitor isolated from tamarind seeds (TTI). * Hematocrit, total leukocyte count, fasting blood glucose and GOT had a non-parametric distribution, so the Kruskall–Wallis test was performed with Dunn’s post-hoc test to verify differences between the pairs. The other parameters presented a parametric distribution, being performed one-way ANOVA test with Tukey’s post-hoc test. Values are presented as mean (standard deviation). Equal lowercase letters on the same line: means did not differ significantly. OB: Obesity; HGLI: high glycemic index and high glycemic load diet. HDL-c: High-density lipoprotein cholesterol; LDL-c: Low-density lipoprotein cholesterol; VLDL-c: Very low-density lipoprotein cholesterol; GOT: Glutamic-oxalacetic transaminase; GPT: Glutamic-pyruvic transaminase; GGT: Gamma glutamyltransferase.

## Data Availability

The data presented in this study are available on request from the corresponding author.

## Supplementary Materials

The following supporting information can be downloaded at: https://www.mdpi.com/article/10.3390/nu14214669/s1, Figure S1: Evaluation of inflammation induction of co-cultures of Caco-2:HT29-MTX cells with 50 ng/mL of TNF-α, on the basolateral side.
