# Supplementary material for: Anti-Inflammatory Protein Isolated from Tamarind Promotes Better Histological Aspects in the Intestine Regardless of the Improvement of Intestinal Permeability in a Preclinical Study of Diet-Induced Obesity
Source: Nutrients. 2022 Nov 4;14(21):4669. doi: 10.3390/nu14214669 (PMC9655259; doi:10.3390/nu14214669)
Supplement: Supplementary file 1 [file nutrients-14-04669-s001.zip › nutrients-1991248-supplementary.pdf]

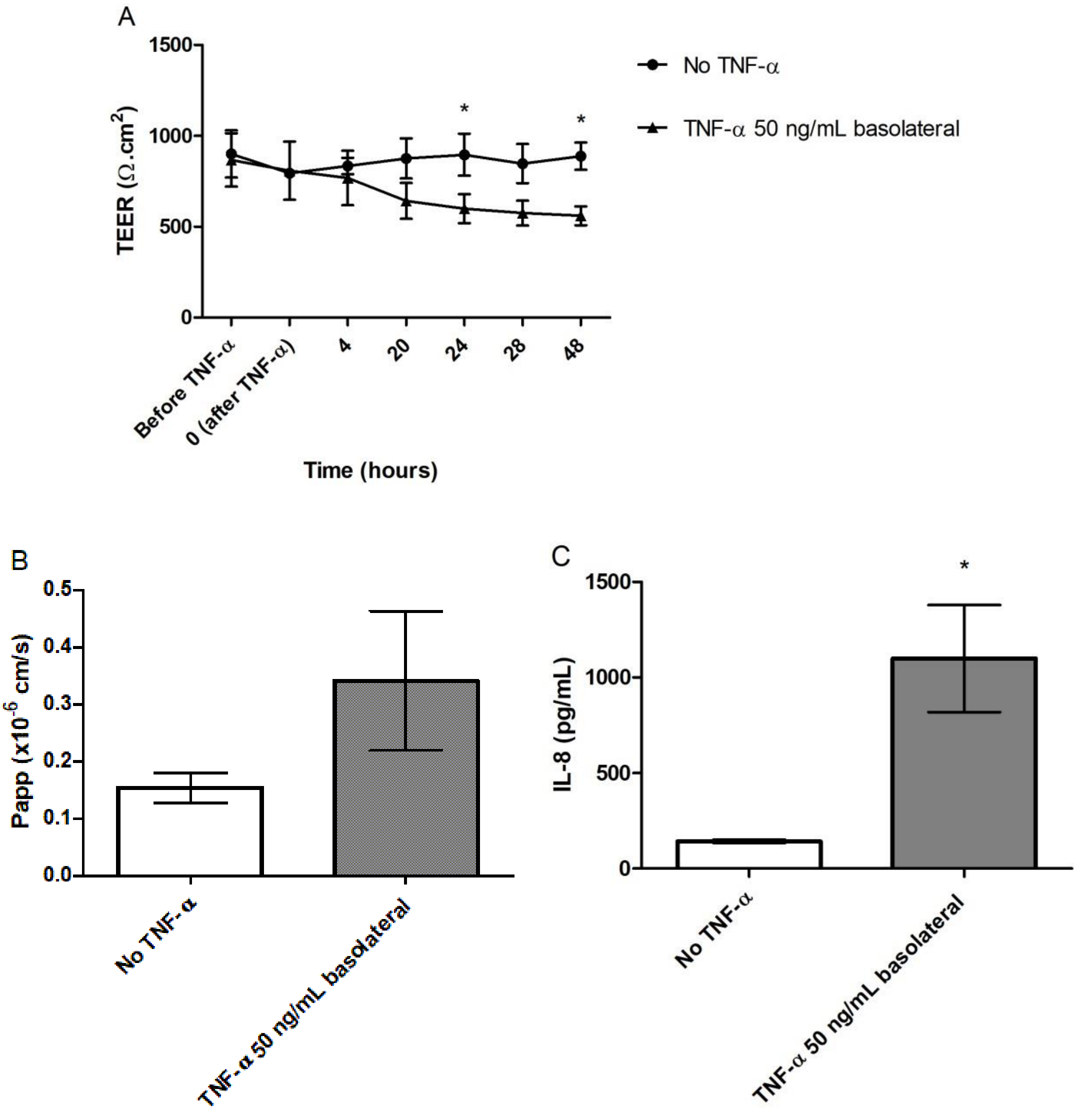

Figura S1. Evaluation of inflammation induction of co-cultures of Caco-2:HT29-MTX cells with 50 ng/mL of TNF-α, on the basolateral side. (A) Transepithelial electrical resistance (TEER) of monolayers over 48 hours of inflammation induction. In this previous test, TEER was reduced by 35% 48h after stimulation with TNF; * $p < 0.05$, 2-way ANOVA with Bonferroni post-hoc test. (B) Apparent permeability of Lucifer Yellow (LY) through cell monolayers after 48 hours of induction of inflammation; $p > 0.05$, t test for unpaired samples and (C) ELISA IL-8 quantification on the basolateral medium after 48 hours of inflammation induction, * $p < 0.05$, t test for unpaired samples.
